# The Influence of the Solid Solution Formation on Purification of *L*-Menthol from the Enantiomer Mixture by Three-Phase Crystallization

**DOI:** 10.3390/ijms241914933

**Published:** 2023-10-05

**Authors:** Yu-Chao Hsu, Sheng-Chin Yang, Kai-Fang Ku, Lie-Ding Shiau

**Affiliations:** 1 Department of Urology, Linkou Chang Gung Memorial Hospital, Taoyuan 333, Taiwan; hsuyc@cgmh.org.tw; 2 Department of Chemical and Materials Engineering, Chang Gung University, Taoyuan 333, Taiwan; nba33150034@gmail.com (S.-C.Y.); cfchu069@gmail.com (K.-F.K.)

**Keywords:** crystallization, vaporization, purification, menthol, thermodynamics process

## Abstract

Three-phase crystallization (TPC) was introduced in this study to purify *L*-menthol from menthol enantiomer mixtures in consideration of the formation of solid solutions. TPC is a new separation technology, which combines melt crystallization and vaporization to result in the desired crystalline product from a liquid mixture along with the unwanted components vaporized via the three-phase transformation by reducing temperature and pressure. The three-phase transformation conditions for the liquid menthol enantiomer mixtures were determined based on the thermodynamic calculations to direct the TPC experiments. A new model was proposed based on the mass and energy balances in consideration of the formation of the solid solutions to predict the yield and purity of the final *L*-menthol product during TPC. The yield and purity obtained from the TPC experiments were compared with those predicted by the model.

## 1. Introduction

Menthol is widely used in perfumery, cigarettes, cough drops, and nasal inhalers for its cooling effect and refreshing flavor [1]. Only *L*-menthol has been reported to have an analgesic effect, whereas *D*-menthol has a lack of an analgesic effect and flavor properties [2,3]. Although natural *L*-menthol is obtained by distillation from the leaves of various subspecies of mint, menthol can also be synthesized via a number of routes, by which a racemic mixture is obtained [4]. Thus, the separation and purification of *L*-menthol from the racemic mixture has been an important research issue. A number of studies have been reported on the bio-catalytic resolution of *L*-menthol from the menthol enantiomer using various lipases [5,6,7,8,9].

Melt crystallization is an important separation process for the purification of organic compounds [10,11,12,13,14,15,16,17,18,19,20,21,22,23,24,25,26,27] and enantiomeric mixtures [28,29]. The unique feature of melt crystallization is that no solvent is required; however, the subsequent separation of formed crystals from melt is often a challenging task for downstream processing. Recently, Shiau and his coworkers have developed a new separation technology, three-phase crystallization (TPC), which combines melt crystallization and vaporization for the separation of the mixtures with the close boiling temperatures [30,31,32,33,34,35,36,37,38]. Basically, TPC is operated via a series of three-phase transformations occurring in a liquid mixture, resulting in the formation of a solid product and vapor. If nearly all the liquid mixture disappears at the end, the final product only consists of solid crystals while the produced vapor can be condensed and collected. Thus, no solid/liquid separation and crystal washing are required at the end of TPC.

Although a pure crystal of the major component is usually obtained in melt crystallization, a solid solution enriched with the major component might be formed for some systems. A solid solution is a solid mixture containing a minor component uniformly distributed within the crystal lattice of the major component, which has never been encountered during TPC in the previous studies [30,31,32,33,34,35,36,37,38]. However, the formation of solid solutions has been reported in melt crystallization for mixtures enriched with *L*-menthol or *D*-menthol enantiomers [39]. In the present study, TPC was applied to purify *L*-menthol from mixtures enriched with *L*-menthol enantiomers in consideration of the formation of solid solutions.

## 2. Principle of TPC

**Figure 1:**
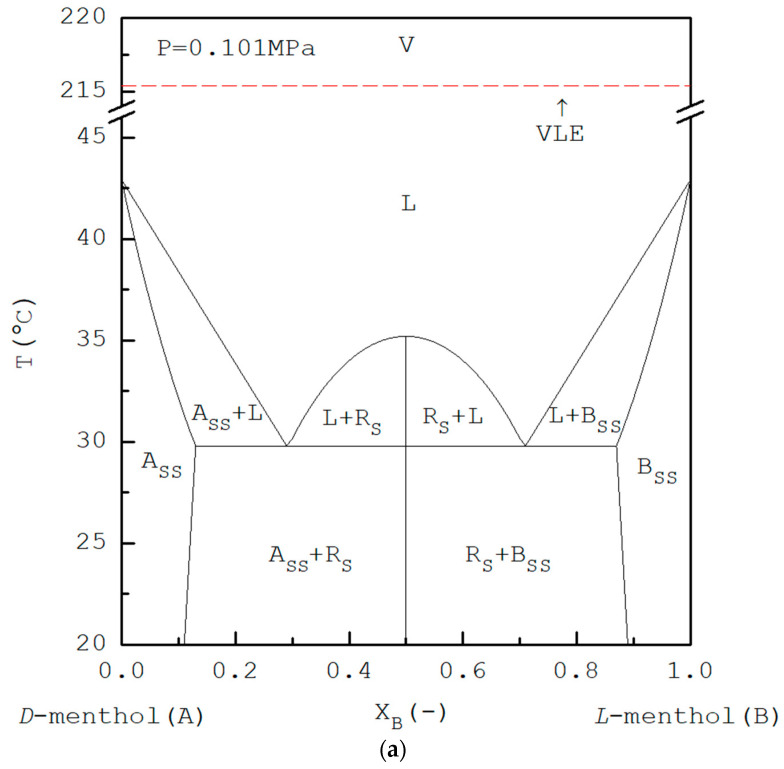

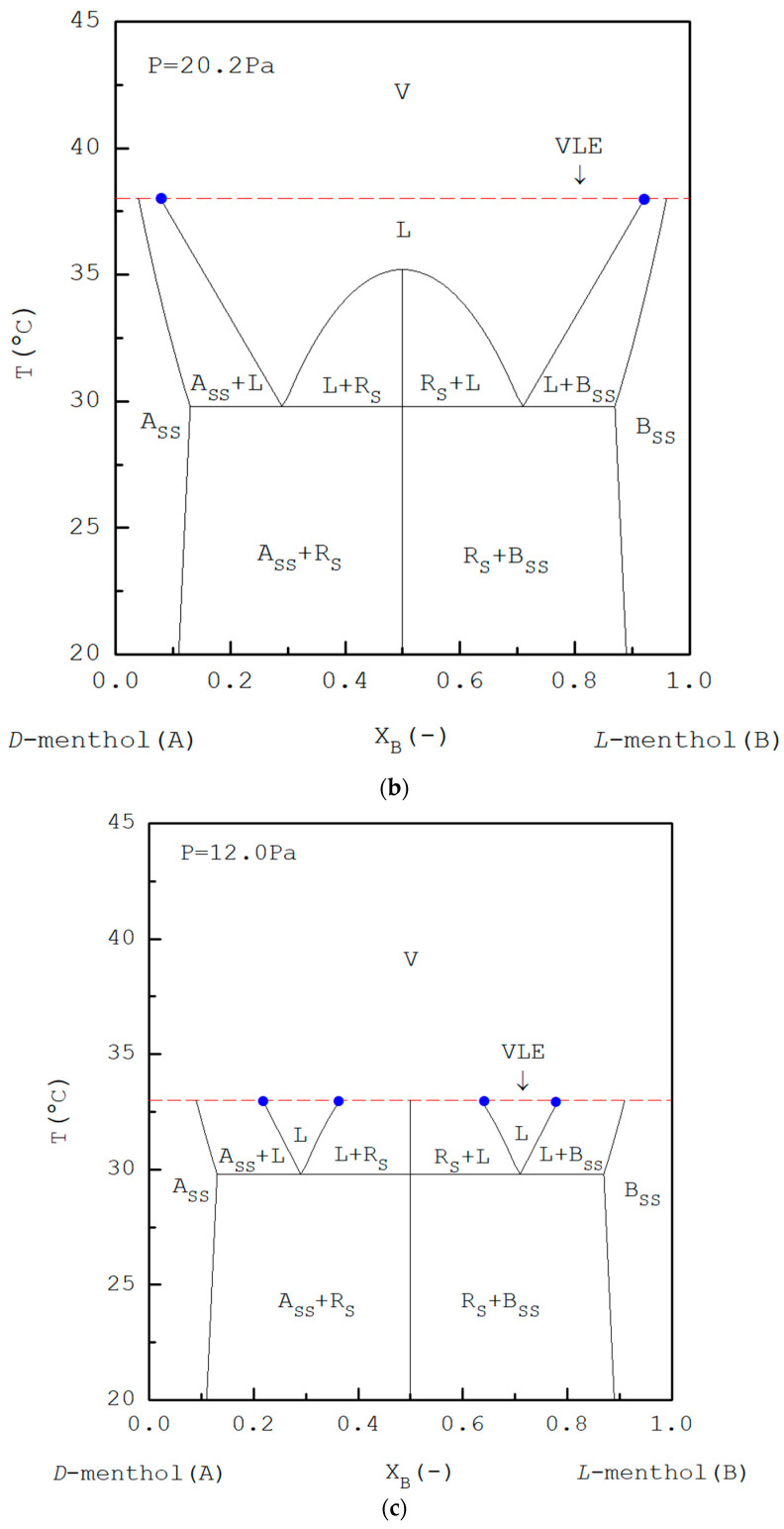
(**a**) The experimental SLE [39] and ideal VLE phase diagram for *D*-menthol and *L*-menthol at P=0.101 MPa, where A_SS_ and B_SS_ represent the solid solution of *D*-menthol and *L*-menthol, respectively, while R_S_ represents the solid racemic compound. (**b**) The predicted SLVE pseudo phase diagram for *D*-menthol and *L*-menthol at P=20.2 Pa. The solid circles represent the predicted three-phase states. (**c**) The predicted SLVE pseudo phase diagram for *D*-menthol and *L*-menthol at P=12.0 Pa. The solid circles represent the predicted three-phase states.

The basic principles of the TPC process can be explained by referring to the phase diagrams. In Figure 1a, the lower part illustrates the experimental solid–liquid equilibrium (SLE) phase diagram for *D*-menthol (A-component) and *L*-menthol (B-component) reported by Corvis et al. [39], where a racemic *DL*-menthol compound is formed at $X_{B}=0.50$ with *T_m_* = 35.2 °C while two eutectic points exist at *T_eu_* = 29.8 °C, one at $X_{B}=0.29$ and the other at $X_{B}=0.71$. The solid solutions enriched with *L*-menthol could be formed in the range $0.71<X_{B}<1$ while the solid solutions enriched with *D*-menthol could be formed in the range $0<X_{B}<0.29$. Although some metastable phases might be formed after recrystallization induced by the thermal quenching of the menthol mixture followed by the heating process [39], this phenomenon should not play an important role during TPC. Some physical properties of *D*-menthol and *L*-menthol are listed in Table 1. Based on vapor pressures measured by Stejfa et al. [40], the temperature-dependent vapor pressure for *L*-menthol is nearly the same as that for *DL*-menthol. The upper part in Figure 1a illustrates the predicted vapor–liquid equilibrium (VLE) at normal pressure, where the equilibrium liquid line coincides with the equilibrium vapor line due to the same saturated vapor pressure assumed for *D*-menthol and *L*-menthol.

Although the melting temperature is generally not influenced by pressure, the boiling temperature is usually decreased with decreasing pressure. Thus, as pressure is reduced, SLE is generally assumed to remain almost the same while the VLE will be moved downward. For example, Figure 1b illustrates the solid–liquid–vapor equilibrium (SLVE) pseudo phase diagram at 20.2 Pa, which is below the triple-point pressure of *D*-menthol and *L*-menthol. Note that the three-phase state occurs at $T_{tri}=42.9\;{}^{\circ}C$ and $P_{tri}=33.9\;Pa$ for *D*-menthol and *L*-menthol while the three-phase state occurs at $T_{tri}=35.2\;{}^{\circ}C$ and $P_{tri}=16.9\;Pa$ for *DL*-menthol [40]. It leads to the existence of two three-phase states at 38.0 °C for the liquid mixture. The first one is a three-phase state having the solid solution of *L*-menthol with $Z_{B}=0.96$, liquid mixture with $X_{B}=0.89$, and vapor mixture with $X_{B}=0.89$ on the right hand side, while the second one is a three-phase state having the solid solution of *D*-menthol with $Z_{B}=0.04$, liquid mixture with $X_{B}=0.11$, and vapor mixture with $X_{B}=0.11$ on the left hand side. Note that, as the solid–vapor equilibrium data are not available in the literature, the solid–vapor equilibrium (SVE) line is not shown here.

As pressure is further reduced, for example, Figure 1c illustrates the SLVE pseudo phase diagram at 12.0 Pa, which is below the triple-point pressure of *DL*-menthol, this leads to the existence of four three-phase states at 33.0 °C for the liquid mixture. The first one is a three-phase state having the solid solution of *L*-menthol with $Z_{B}=0.91$, liquid mixture with $X_{B}=0.78$, and vapor mixture with $X_{B}=0.78$; the second one is a three-phase state having the *DL*-menthol solid liquid mixture with $X_{B}=0.64$ and vapor mixture with $X_{B}=0.64$; the third one is a three-phase state having the *DL*-menthol solid liquid mixture with $X_{B}=0.36$ and vapor mixture with $X_{B}=0.36$; and the fourth one is a three-phase state having the solid solution of *L*-menthol with $Z_{B}=0.09$, liquid mixture at $X_{B}=0.22$, and vapor mixture at $X_{B}=0.22$. It should be noted in Figure 1b,c that only the first three-phase state on the right-hand side can be encountered for the liquid mixture in the range $0.71<X_{B}<1$ during TPC, leading to the formation of the solid solution of *D*-menthol. In other words, as other three-phase states cannot be achieved for the liquid mixture in the range $0.71<X_{B}<1$ during TPC, the *DL*-menthol solid or the solid solution of *L*-menthol cannot be formed.

## 3. TPC Model

**Figure 2:**
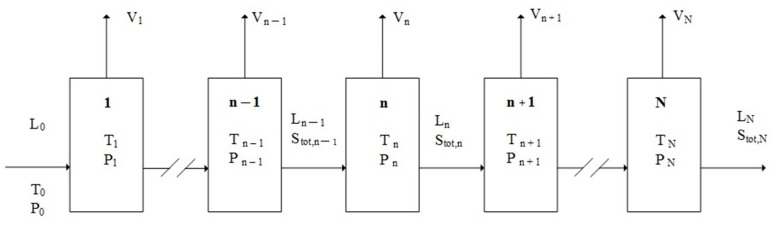
Schematic diagram of the TPC operation where each stages is operated at a three-phase transformation state.

If *L*-menthol is in the range $0.71<X_{B}<1$ in the liquid mixture of *D*-menthol and *L*-menthol, TPC can be applied to produce the crystalline product of the solid solutions enriched with *L*-menthol along with a mixture vapor from the liquid mixture. The TPC process starts with a liquid mixture and can be simulated in a series of stage operations shown in Figure 2, where each stage is operated at the three-phase transformation condition. Consequently, both melt crystallization and vaporization occur in the liquid mixture in each stage, resulting in the formation of the solid solution of *L*-menthol along with the vapor. The vapor formed in each stage is removed while the solid formed and the liquid remaining in each stage enter the next stage.

As each stage is operated at the three-phase transformation condition, both the SLE and VLE need to be satisfied in each stage. According to the SLE data in Figure 1 measured by Corvis et al. [39], the relationship between $T_{n}$ and the concentration of *L*-menthol in the mixture liquid ${{(X}_{B})}_{n}$ for the SLE in stage *n* is fitted as:

(1) $$T_{n}=45.17{{2(X}_{B})}_{n}+270.88\;$$

In consideration of the formation of the solid solutions, the relationship between $T_{n}$ and the concentration of *L*-menthol in the solid solution ${{(Z}_{B})}_{n}$ for the SLE in stage *n* is fitted as:

(2) $$T_{n}=93.936{{(Z}_{B})}_{n}+220.704\;\;\;$$

Due to low pressures, the ideal gas law is assumed for the mixture vapor. The VLE for each component in stage *n* can be described by Raoult’s law as [41,42]:

(3) $${{(Y}_{A})}_{n}P_{n}={{(X}_{A})}_{n}{{(\gamma }_{A})}_{n}{{(P}_{A}^{sat})}_{n}\;\;$$

(4) $${{(Y}_{B})}_{n}P_{n}={{(X}_{B})}_{n}{{(\gamma }_{B})}_{n}{{(P}_{B}^{sat})}_{n}$$

where ${{(\gamma }_{A})}_{n}={{(\gamma }_{B})}_{n}=1$ is assumed due to the structure similarity between *D*-menthol and *L*-menthol. Note that ${{(X}_{A})}_{n}+{{(X}_{B})}_{n}=1$ and ${{(Y}_{A})}_{n}+{{(Y}_{B})}_{n}=1$.

Based on the experimental data measured by Stejfa et al. [40], the temperature dependence of the saturated vapor pressure of *L*-menthol is fitted as:

(5) $${{(P}_{B}^{sat})}_{n}=exp\left(35.211-\frac{10,015}{T_{n}}\right)\;\;$$

where ${{(P}_{B}^{sat})}_{n}$ is in Pa and $T_{n}$ is in K. As ${{(P}_{A}^{sat})}_{n}={{(P}_{B}^{sat})}_{n}$ is assumed for simplicity, combining Equations (3)–(5) yields:

(6) $${{(Y}_{B})}_{n}={{(X}_{B})}_{n}\;\;\;$$

(7) $$P_{n}={{(P}_{A}^{sat})}_{n}={{(P}_{B}^{sat})}_{n}$$

The initial three-phase transformation condition $\left(T_{0},\;P_{0}\right)$ for the liquid mixture feed with an initial concentration ${\left(X_{B}\right)}_{0}$ can be determined as follows: (a) $T_{0\;}$ is determined for ${\left(X_{B}\right)}_{0}$ by Equation (1); (b) $P_{0\;}$ is determined for $T_{0\;}$ by (7). Once $T_{0\;}$ is determined, $T_{n}$ in each stage can be specified by $T_{n}=T_{n-1}-∆T$ (n=1,2,…,N) for a chosen ∆T. Then, the corresponding pressure $P_{n}$ for the three-phase transformation condition is determined by (7) for each $T_{n}$. Consequently, ${{(X}_{B})}_{n}$ and ${{(Z}_{B})}_{n}$ are determined respectively by Equations (1) and (2) for each $T_{n}$. Thus, if $T_{n}$ is specified in stage *n*, then $P_{n}$, ${{(X}_{A})}_{n}$, ${{(X}_{B})}_{n}$, ${{(Y}_{A})}_{n}$, ${{(Y}_{B})}_{n}$, ${{(Z}_{A})}_{n}$, and ${{(Z}_{B})}_{n}$ for the corresponding three-phase transformation condition can be determined as described above, which is consistent with the phase rule defined by F=C+2−Φ [41,42]. As this system consists of two components in a series of three-phase transformations, one obtains F=1 due to C=2 and Φ=3 in each stage.

**Figure 3:**
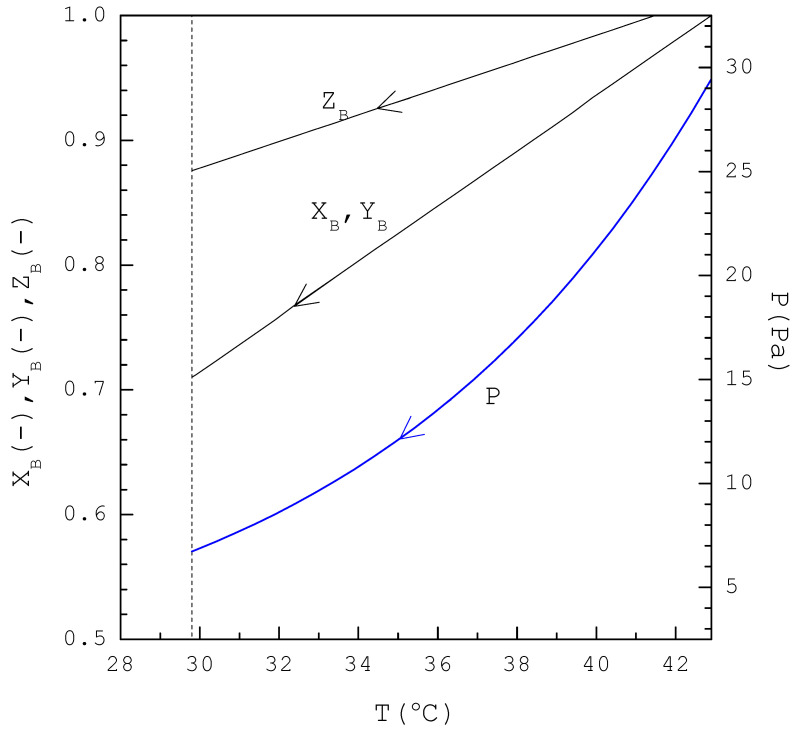
The calculated results of P(T), $X_{B}(T)$, $Y_{B}\left(T\right)$, and $Z_{B}\left(T\right)$ for the three-phase transformation during the TPC cooling process.

Figure 3 displays the variations of P(T), $X_{B}(T)$, $Y_{B}\left(T\right),$ and $Z_{B}(T)$ during the TPC cooling process. Thus, P, $X_{B}$, $Y_{B}$, and $Z_{B}$ decreases as temperature decreases. The TPC process is generally stopped at the eutectic temperature (29.8 °C) when all the liquid is solidified. If the pressure in each stage is controlled according to P(T) in Figure 3 during the cooling process, the three-phase transformation occurs in each stage.

If TPC is started with a liquid mixture $L_{0}$ with the initial concentration ${(X}_{B}{)}_{0}$, the variations of $L_{n}$, $S_{n}$, and $V_{n}$ in stage *n* can be derived as follows, where $L_{n}$ represents the amount of liquid mixture remaining in stage *n*, $S_{n}$ represents the amount of solid crystalline product formed in stage *n*, and $V_{n}$ represents the amount of vapor mixture formed in stage *n*. It should be noted that, as shown in Figure 2, $V_{n}$ formed in each stage is removed from the vessel to keep the pressure operated at the three-phase transformation pressure $P_{n}$ while $L_{n}$ and $S_{n}$ in each stage are kept in the vessel and enter the next stage.

As $L_{n-1}-L_{n}$ represents the amount of liquid mixture that disappeared in stage *n*, it leads to the formation of solid crystalline product ${(S}_{n})$ and vapor mixture $\left(V_{n}\right)$ in stage *n*. The total material balance in stage *n* can be written as:

(8) $$L_{n-1}-L_{n}=S_{n}+V_{n}$$

The material balance of *L*-menthol in stage *n* can be written as:

(9) $$L_{n-1}{{(X}_{B})}_{n-1}-L_{n}{{(X}_{B})}_{n}=S_{n}{{(Z}_{B})}_{n}+V_{n}{{(Y}_{B})}_{n}$$

It was observed during the experiments that both melt crystallization and vaporization occurred in the liquid mixture very quickly in each stage, leading to the formation of the crystalline product and the vapor mixture. As the liquid mixture ${(L}_{n-1}-L_{n})$ is simultaneously crystallized and vaporized due to the three-phase transformation in stage *n*, it is assumed that the vaporization heat required to form the vapor mixture ${(V}_{n})$ is provided by the crystallization heat released in forming the solid crystalline product ${(S}_{n})$ in stage *n*. Thus, the energy balance in stage *n* is given by:

(10) $$S_{n}\left[{{{(Z}_{A})}_{n}∆H}_{m,A}+{{{(Z}_{B})}_{n}∆H}_{m,B}\right]=V_{n}\left[{{{(Y}_{A})}_{n}∆H}_{V,A}+{{{(Y}_{B})}_{n}∆H}_{V,B}\right]$$

Due to the structure similarity between *D*-menthol and *L*-menthol, it is assumed that ${∆H}_{m,A}={∆H}_{m,B}$ and ${∆H}_{V,A}={∆H}_{V,B}$. Equation (10) reduces to:

(11) $$S_{n}{∆H}_{m,B}=V_{n}{∆H}_{V,B}$$

If the feed is a liquid mixture only, one has $L_{0}$ with an initial concentration ${\left(X_{B}\right)}_{0}$, leading to $S_{0}=V_{0}=0$. To solve for $L_{n}$, $S_{n}$, and $V_{n}$ in stage n (n=1,2,…,N), combining Equations (8) and (9) due to ${{(Y}_{B})}_{n}={{(X}_{B})}_{n}$ yields:

(12) $$S_{n}=\frac{L_{n-1}\left[{\left(X_{B}\right)}_{n-1}-{\left(X_{B}\right)}_{n}\right]}{{\left(Z_{B}\right)}_{n}-{\left(X_{B}\right)}_{n}}$$

where ${{(X}_{B})}_{n}$ and ${\left(X_{B}\right)}_{n-1}$ are determined from Equation (1) for $T_{n}$ and $T_{n-1}$, respectively, while ${{(Z}_{B})}_{n}$ is determined from Equation (2) for $T_{n}$. As $L_{n-1}$ is known, $S_{n}$ can be determined from Equation (12); and subsequently,$\;V_{n}$ can be determined from Equation (11) and $L_{n}$ can be determined from Equation (8).

As shown in Figure 2, $S_{tot,n}=\sum_{j=1}^{n}S_{j}$ represents the total amount of crystalline product formed from stage 1 to stage *n*. Thus, $L_{n}$ and $S_{tot,n}$ exit from stage *n* and enter stage *n* + 1 while $V_{n}$ formed in stage *n* is removed from the system and does not enter stage *n* + 1. Note that $L_{N}$ represents the amount of liquid mixture remaining at the end of the TPC. By definition, $S_{tot,N}=\sum_{j=1}^{N}S_{j}$ represents the total amount of crystalline product at the end of TPC while $V_{tot,N}=\sum_{j=1}^{N}V_{j}$ represents the total amount of vapor mixture formed and removed from stage 1 to stage *N* during TPC. Thus, $S_{tot,N}$ and $L_{N}$ are obtained as the final product in the last stage at the end of TPC.

## 4. Experimental Section

**Figure 4:**
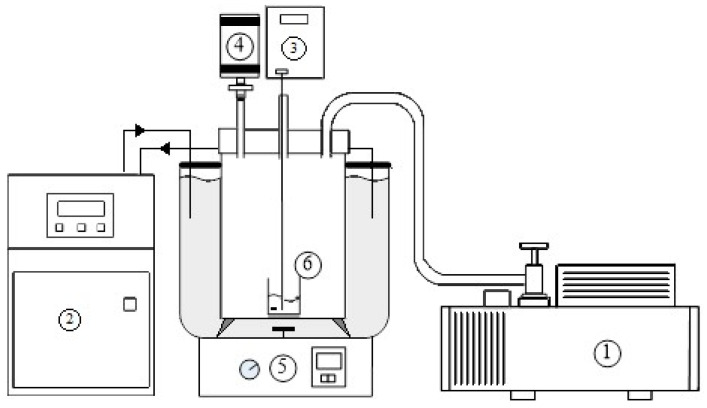
Schematic diagram of the experimental apparatus for TPC with the features: (1) mechanical vacuum pump, (2) thermostat, (3) thermocouple, (4) pressure gauge, (5) magnetic stirrer, and (6) sample container.

The experimental assembly consisted of a 50-mL sample container in a 1.2-L stainless vessel with the transparent cover on top shown in Figure 4. The stainless vessel was immersed in water for temperature control and connected to a mechanical vacuum pump for pressure control. *D*-menthol (purity > 99.0%) was purchased from Tokyo Chemical Industry and *L*-menthol (purity > 99.5%) was purchased from Acros Organics.

In the beginning of the experiment, 5 g of liquid mixture feed with an initial concentration ${\left(X_{B}\right)}_{0}$ was placed in the sample container stirred by a magnetic bar at 70 rpm. A temperature probe was positioned in the feed sample and a pressure gauge was connected to the vessel. The operating temperature and pressure in the stainless vessel during the TPC experiments were adjusted by controlling the thermostat and the mechanical vacuum pump, respectively. Crystallization and vaporization in the liquid mixture due to the three-phase transformation was observed via the transparent cover on top of the vessel.

To perform the batch TPC experiment, the initial three-phase transformation condition $\left(T_{0},\;P_{0}\right)$ for 5 g of liquid mixture feed with a specified ${\left(X_{B}\right)}_{0}$ was determined first. For example, it led to $T_{0}=38.4\;{}^{\circ}C$ and $P_{0}=21.4\;Pa$ for ${\left(X_{B}\right)}_{0}=0.90$. Initially, 5 g of liquid mixture feed with a specified ${\left(X_{B}\right)}_{0}$ was placed in the sample container. Then, the initial three-phase transformation condition $\left(T_{0},\;P_{0}\right)$ was reached for the liquid mixture feed by lowering temperature and pressure at t=0. Once the three-phase transformation condition ${(T}_{0\;},P_{0})$ was reached, temperature was lowered gradually at a cooling rate of 1.0 °C/min and the pressure was adjusted downward according to P(T) in Figure 3.

For simplicity, the batch TPC experiment performed in this work can be illustrated in Figure 2, where each stage corresponds to a three-phase transformation state at a certain time during the batch TPC experiment. Thus, both melt crystallization and vaporization occurred in the liquid mixture in each stage. To ensure that the three-phase transformation occurred and finished for the liquid mixture in each stage, the corresponding temperature and pressure was maintained in each stage for 1 min as the three-phase transformation condition ${(T}_{n\;},P_{n})$ was reached.

For the batch TPC experiment cooled at 1.0 °C/min, as the three-phase transformation condition ${(T}_{1\;},P_{1})$ was reached in stage 1 at *t*_1_, the operating condition was maintained at ${(T}_{1\;},P_{1})$ in stage 1 for 1 min. Consequently, the three-phase transformation occurred in the initial liquid mixture, resulting in some portion of the initial liquid transformed to the solid crystalline product and vapor in stage 1. Only the solid crystalline product and the remaining liquid were contained in stage 1 and entered stage 2 while the vapor was removed from the vessel. Subsequently, as the three-phase transformation condition ${(T}_{2\;},P_{2})$ was reached in stage 2 at *t*_2_

, the operating condition was maintained at ${(T}_{2\;},P_{2})$ in stage 2 for 1 min. Consequently, the three-phase transformation occurred in the remaining liquid, resulting in some portion of the remaining liquid transformed to the solid crystalline product and vapor in stage 2. Only the solid crystalline product and the remaining liquid were contained in stage 2 and entered stage 3 while the vapor was removed from the vessel. Thus, more solid crystalline product and less remaining liquid were obtained in stage 2 than those in stage 1.

Similarly, as TPC was operated by lowering temperature and pressure in each stage, a series of three-phase transformations occurred in the remaining liquid. Consequently, as the stage number increased, the solid crystalline product increased and the remaining liquid decreased. As the vapor formed in each stage was removed from the vessel, the final product only consisted of the solid crystalline product and the remaining liquid.

At the end of the TPC experiments, some crystalline product of the *L*-menthol solid solution along with the remaining liquid was obtained in the sample container. As the TPC experiments were ended at the eutectic temperature (29.8 °C), the remaining liquid was solidified; and subsequently, it was difficult to separate the crystalline product from the remaining liquid, and the yield of the final product $\left(W_{f,exp}\right)$, including the crystalline product and the remaining liquid, in the sample container was weighed. By mixing the crystalline product and the remaining liquid together, the enantiomeric purity of the final product $\left(C_{B,exp}\right)$ was analyzed by dissolving 0.1 g of the final product in a 20 mL ethanol solution using a Polarimeter (model: SEPA-300, Horiba, Japan). As the measured specific optical rotation versus the known enantiomeric purity of the sample was experimentally measured first, the enantiomeric purity of the final product could be determined by measuring its specific optical rotation. Note that ${\left[\alpha \right]}_{D}^{20}=-48.9{}^{\circ}$ for *L*-menthol and ${\left[\alpha \right]}_{D}^{20}=48.9{}^{\circ}$ for *D*-menthol.

Some sweating experiments were further performed on the obtained product from the TPC experiments. The sweating apparatus adopted here was the same as that reported in our previous work [35]. In the sweating experiments, the final product, including the *L*-menthol solid solution and remaining liquid mixture, obtained at the end of TPC was placed on top of the stainless sieve inside the bottom of a glass tube. The glass tube was closed with a lid and kept isothermally in a thermostat bath maintained at 42° for 30 min. The melting takes place preferentially on the solidified mixture liquid due to its lower melting temperature compared to *L*-menthol [12]. Thus, the liquid adhering to the crystal surface and contained in the crystal was discharged through the sieve mesh under the influence of gravity. At the end of the sweating experiments, the product remaining on top of the stainless sieve was weighed and analyzed by polarimetry. Thus, the amount of liquid removed ${(∆L}_{exp}$) and the experimental purity of *L*-menthol for the final product at the end of the sweating experiments (${\bar{Z}}_{B,exp})$ were determined.

## 5. Results and Discussion

TPC was applied to purify *L*-menthol for 5 g of liquid mixture feed ${(L}_{0}=5\;g)$ with $0.90\leq {(X}_{B}{)}_{0}\leq 0.97$. Table 2 lists the calculated results for $L_{0}=5\;g$ with ${(X}_{B}{)}_{0}=0.90$ using ∆T=1 °C for N=9, where $T_{0}=38.4\;{}^{\circ}C$ and $P_{0}=21.4\;Pa$ is the initial three-phase transformation condition for the liquid mixture. As $T_{n}$ is specified in each stage using $T_{n}=T_{n-1}-1$, $P_{n}$ is determined from Equation (7); and ${\left(X_{B}\right)}_{n}$ and ${\left(Z_{B}\right)}_{n}$ are determined, respectively, from Equations (1) and (2). Note that ${\left(Y_{B}\right)}_{n}={\left(X_{B}\right)}_{n}$. Consequently, $S_{n}$, $L_{n}$, and $V_{n}$ are determined as described previously. Table 2 indicates that, as n increases during the cooling process, $S_{tot,n}$ increases and $L_{n}$ decreases. Based on the total material balance, one obtains $L_{0}=S_{tot,n}+V_{tot,n}+L_{n}$ in each stage. If TPC is operated from 38.4 °C and 21.4 Pa (n=1) to 29.4 °C and 8.2 Pa (n=9), it yields $S_{tot,N}=3.8\;g$ and $L_{N}=0.50\;g$ in the last stage. Similarly, the calculated results for $L_{0}=5\;g$ with ${(X}_{B}{)}_{0}=0.93–0.97$ are listed in Tables S1–S3 (see Supplementary Materials).

The batch TPC experiments were performed based on the corresponding T and P in each stage for various ${\left(X_{B}\right)}_{0}$ in Table 2 and Tables S1–S3. The TPC experiments were started from the initial three-phase transformation condition ${(T}_{0},\;P_{0})$ determined for each ${(X}_{B}{)}_{0}$ and were ended at the final three-phase transformation condition (29.8 °C,8.5 Pa). It was found that vaporization was first observed when the initial three-phase transformation condition was reached; and as the final three-phase transformation condition was reached, all the liquid was solidified and no vaporization was observed. The experiments revealed that a series of three-phase transformations generally occurred very fast during TPC. Each batch TPC experiment generally finished at around 25 min at a cooling rate of 1.0 °C/min as the temperature was decreased from the melting point (42.9 °C) to the eutectic temperature (29.8 °C).

**Figure 5:**
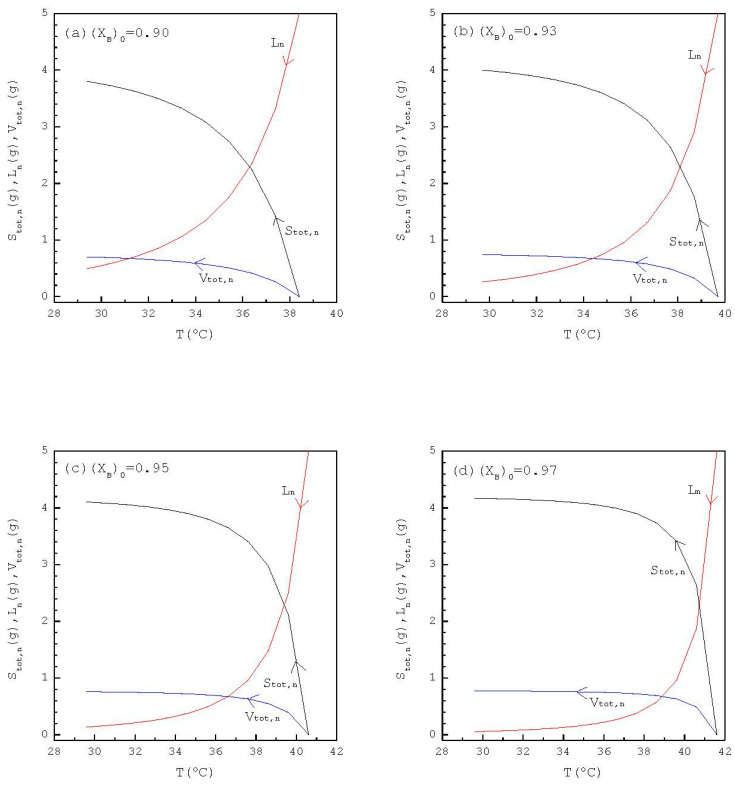
The calculated results of $L_{n}$, $S_{tot,n}$, and $V_{tot,n}$ in each stage during the TPC cooling process for $L_{0}=5g$ with (**a**) ${\left(X_{B}\right)}_{0}=0.90$; (**b**) ${\left(X_{B}\right)}_{0}=0.93$; (**c**) ${\left(X_{B}\right)}_{0}=0.95$; (**d**) ${\left(X_{B}\right)}_{0}=0.97$.

Figure 5 shows the calculated results of $L_{n}$, $S_{tot,n}$, and $V_{tot,n}$ for $L_{0}=5\;g$ with various ${\left(X_{B}\right)}_{0}$ listed in Table 2 and Tables S1–S3. It should be note that $L_{n}$ for ${\left(X_{B}\right)}_{0}=0.97$ decreases more rapidly during the early cooling process than that for ${\left(X_{B}\right)}_{0}=0.90$; and, subsequently, $S_{tot,n}$ for ${\left(X_{B}\right)}_{0}=0.97$ increases more rapidly during the early cooling process than that for ${\left(X_{B}\right)}_{0}=0.90$.

According to the calculated results in Table 2 and Tables S1–S3, some liquid remained with the final *L*-menthol solid solution at the end of TPC. As the final product consists of the *L*-menthol solid solution and remaining liquid, the theoretical yield for the final product at the end of TPC is given by:

(13) $$W_{f,the}=L_{N}+\sum_{j=1}^{N}S_{j}=L_{N}+S_{tot,N}$$

**Figure 6:**
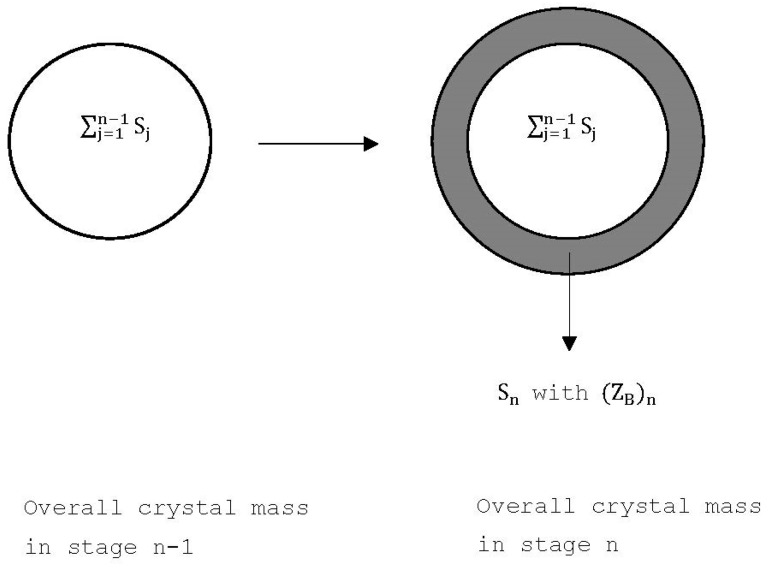
Incremental change in the crystal mass from $\sum_{j=1}^{n-1}S_{j}$ in stage n−1 to $\sum_{j=1}^{n}S_{j}$ in stage n during the TPC cooling process, where $S_{n}$ for the shaded area represents the solid crystalline product formed in stage *n* and ${{(Z}_{B})}_{n}$ represents the concentration of L-menthol for the solid crystalline product formed in stage *n*.

As illustrated in Figure 6, the amount of solid solution formed in stage *n* is $S_{n}$ with the purity ${{(Z}_{B})}_{n}$ due to the formation of the solid solution during TPC. As the total amount of solid solution formed during TPC is $\sum_{j=1}^{N}S_{j}$ and the total amount of *L*-menthol in the final solid solution at the end of TPC is $\sum_{j=1}^{N}S_{j}{{(Z}_{B})}_{j}$, the theoretical purity of *L*-menthol for the final crystalline product without the remaining liquid at the end of TPC is given by:

(14) $${\bar{Z}}_{B,the}=\frac{\sum_{j=1}^{N}S_{j}{{(Z}_{B})}_{j}}{\sum_{j=1}^{N}S_{j}}$$

As the final product consists of the *L*-menthol solid solution and remaining liquid, the theoretical purity of *L*-menthol for the final product at the end of TPC is given by:

(15) $$C_{B,the}=\frac{{L_{N}(X_{B})}_{N}+\sum_{j=1}^{N}S_{j}{{(Z}_{B})}_{j}}{L_{N}+\sum_{j=1}^{N}S_{j}}$$

**Figure 7:**
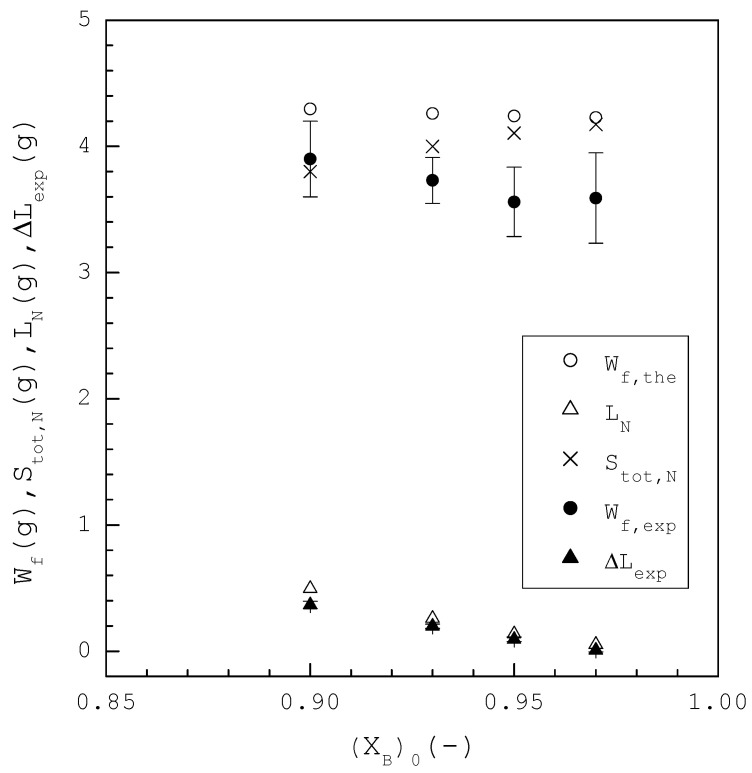
Comparison of $W_{f,the}$, $\;W_{f,exp}$, $S_{tot,N}$, $L_{N}$, and ${∆L}_{exp}$ for each ${\left(X_{B}\right)}_{0}$, where three repetitive experiments were performed for each ${\left(X_{B}\right)}_{0}$ and error bars represent the 95% confidence intervals for the experimental $W_{f,exp}$ or ${∆L}_{exp}$.

**Figure 8:**
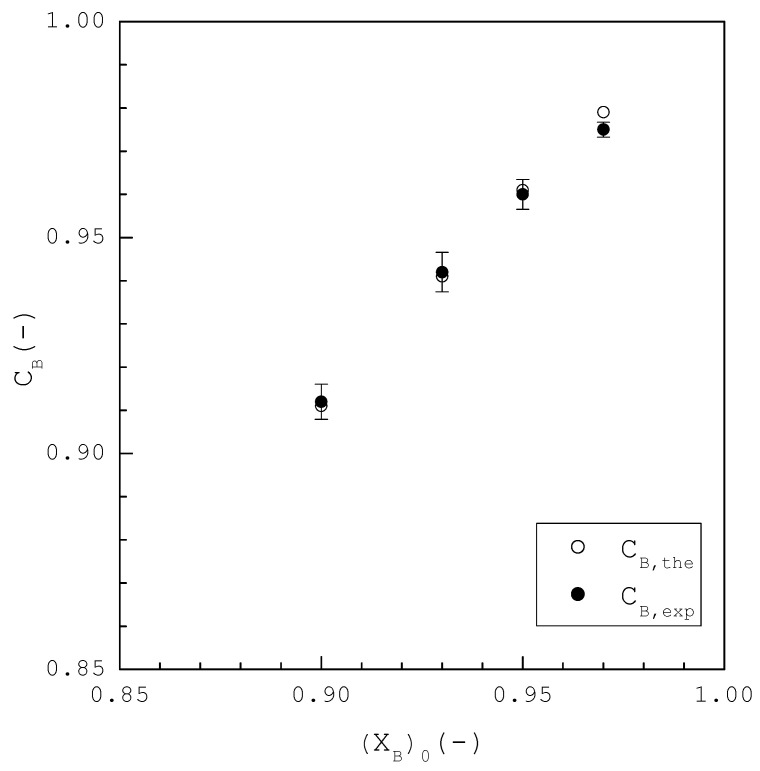
Comparison between $C_{B,the}$ and $C_{B,exp}$ at the end of the TPC experiments for each ${\left(X_{B}\right)}_{0}$, where three repetitive experiments were performed for each ${\left(X_{B}\right)}_{0}$ and error bars represent the 95% confidence intervals for the experimental $C_{B,exp}$.

Figure 7 shows a comparison of $W_{f,the}$,$\;W_{f,exp}$, $S_{tot,N}$, $L_{N}$, and ${∆L}_{exp}$ for each ${\left(X_{B}\right)}_{0}$, where three repetitive experiments were performed for each ${\left(X_{B}\right)}_{0}$ and error bars represent the 95% confidence intervals for the experimental $W_{f,exp}$ or ${∆L}_{exp}$. Note that $W_{f,exp}$ represents the experimental yield of the final product, including the crystalline product and the remaining liquid at the end of TPC while ${∆L}_{exp}$ represents the amount of liquid removed by sweating. As ${\left(X_{B}\right)}_{0}$ increases from 0.90 to 0.97, $L_{N}$ decreases from 0.495 g to 0.054 g while $S_{tot}$ increases from 3.80 g to 4.17 g. Consequently, $W_{f,the}$ remains nearly the same in the range of 4.23 g to 4.30 g. It should be noted that, for a higher ${\left(X_{B}\right)}_{0}$, $L_{N}$ decreases while $S_{tot,N}$ increases at the end of TPC; and, consequently, as shown in Figure 8, a higher ${\left(X_{B}\right)}_{0}$ leads to a higher $C_{B,the}$ due to a smaller $L_{N}$ in the final product. The TPC experiments indicated that $W_{f,exp}$ is in the range of 3.56 g to 3.90 g, which is generally smaller than $W_{f,the}$. The sweating experiments indicated that ${∆L}_{exp}$ is in the range of 0.026 g to 0.36 g, which is slightly smaller than $L_{N}$ due to some liquid still remaining in the final product at the end of sweating.

Figure 8 shows $C_{B,the}$ and $C_{B,exp}$ of the final product at the end of TPC plotted against various ${\left(X_{B}\right)}_{0}$. For example, ${(X}_{B}{)}_{0}=0.90$ was experimentally purified to $C_{B,exp}=0.912$ by TPC, as opposed to $C_{B,the}=0.910$ while ${(X}_{B}{)}_{0}=0.97$ was experimentally purified to $C_{B,exp}=0.975$ by TPC, as opposed to $C_{B,the}=0.978$. Thus, $C_{B,exp}$ is generally close to $C_{B,the}$ for each ${\left(X_{B}\right)}_{0}$.

**Figure 9:**
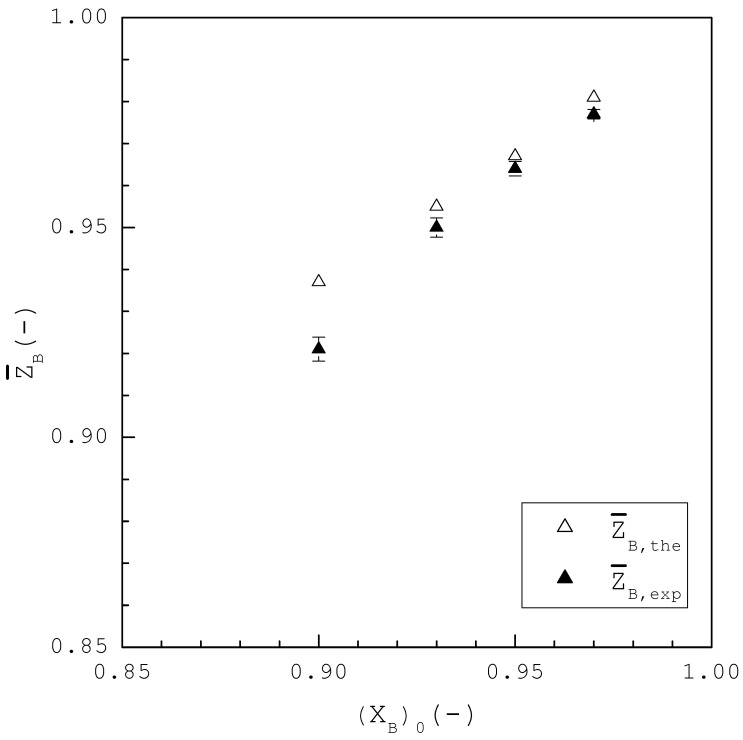
Comparison between ${\bar{Z}}_{B,the}$ and ${\bar{Z}}_{B,exp}$ at the end of the sweating experiments for each ${\left(X_{B}\right)}_{0}$, where three repetitive experiments were performed for each ${\left(X_{B}\right)}_{0}$ and error bars represent the 95% confidence intervals for the experimental ${\bar{Z}}_{B,exp}$.

Figure 9 shows ${\bar{Z}}_{B,the}$ and ${\bar{Z}}_{B,exp}$ of the final product at the end of sweating plotted against various ${\left(X_{B}\right)}_{0}$, where ${\bar{Z}}_{B,exp}$ represents the experimental purity of L-menthol for the final product at the end of sweating while, as defined in Equation (14), ${\bar{Z}}_{B,the}$ represents the theoretical purity of L-menthol for the final product at the end of sweating if all the liquid is removed. For example, ${(X}_{B}{)}_{0}=0.90$ was experimentally purified to ${\bar{Z}}_{B,exp}=0.921$ by sweating, as opposed to ${\bar{Z}}_{B,the}=0.937$ while ${(X}_{B}{)}_{0}=0.97$ was experimentally purified to ${\bar{Z}}_{B,exp}=0.977$ by sweating, as opposed to ${\bar{Z}}_{B,the}=0.981$. Thus, as some liquid was still remaining in the final product at the end of sweating, ${\bar{Z}}_{B,exp}$ is slightly smaller than ${\bar{Z}}_{B,the}$ for each ${\left(X_{B}\right)}_{0}$. By comparing Figure 8 and Figure 9, ${\bar{Z}}_{B,exp}$ at the end of sweating is greater than $C_{B,exp}$ at the end of TPC for each ${\left(X_{B}\right)}_{0}$. Thus, as some liquid was removed by sweating, the purity of the final product obtained by TPC was further increased by sweating.

## 6. Conclusions

In consideration of the formation of the solid solutions, TPC was successfully applied for chiral purification of L-menthol from the menthol enantiomer mixture with the initial concentrations of L-menthol greater than 0.90. The three-phase transformation conditions determined from the SLE and saturated vapor pressure reported in the literature were employed to direct the TPC experiments. By taking the formation of L-menthol solid solution into account, a new model was proposed based on the mass and energy balances to predict the yield and purity of the final L-menthol product during the TPC experiments. The TPC experiments indicated that, although the experimental yield of the final L-menthol product was slightly lower than that predicted by the model, the experimental purity of the final L-menthol product was consistent with that predicted by the model. The sweating experiments can be further performed on the obtained product from the TPC experiments to remove the remaining liquid adhering to the crystal surface and contained in the crystal. Although the increase in the chiral purity was quite limited due to the formation of the solid solution, TPC provides an alternative method for the purification of L-menthol from the enantiomer mixture. In practical applications, L-menthol could be more efficiently purified if consecutive TPC processes are applied, e.g., the final L-menthol crystalline product from the first batch process is melt and used as the liquid feed in the second batch process for further purification, etc. As opposed to melt crystallization at atmospheric pressure, no solid/liquid separation and crystal washing are required at the end of TPC if nearly all the liquid mixture is vaporized during TPC.

## Figures and Tables

**Table 1:** Some physical properties for menthol [40].

Property	*L*-Menthol (*D*-Menthol)
Molecular structure	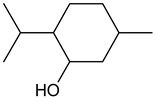
Molecular weight	156.27
T_b_ (°C)	215.4
T_m_ (°C)	42.9
P_tri_ (Pa)	33.9
∆H_m_ (J/mol)	1.347 × 10^4^
∆H_v_ (J/mol)	7.28 × 10^4^

**Table 2:** The calculated results of TPC for *L*_0_ = 5 g feed with (*X_B_*)_0_ = 0.90 (∆*T* = 1 °C).

*n*	*T* (°C)	*P* (Pa)	*(X_B_)n*	*(Z_B_)n*	*L_n_* (g)	*S_n_* (g)	*S_tot,n_* (g)	*V_n_* (g)	*V_tot,n_* (g)
0	38.4	21.4	0.9	0.967	5	0	0	0	0
1	37.4	19.3	0.878	0.956	3.326	1.412	1.412	0.261	0.261
2	36.4	17.4	0.856	0.946	2.355	0.819	2.232	0.152	0.413
3	35.4	15.7	0.834	0.935	1.746	0.514	2.746	0.095	0.508
4	34.4	14.1	0.812	0.924	1.34	0.342	3.089	0.063	0.571
5	33.4	12.7	0.789	0.914	1.057	0.239	3.327	0.044	0.615
6	32.4	11.4	0.767	0.903	0.853	0.172	3.499	0.032	0.647
7	31.4	10.2	0.745	0.892	0.701	0.128	3.628	0.024	0.671
8	30.4	9.2	0.723	0.882	0.585	0.098	3.725	0.018	0.689
9	29.4	8.2	0.701	0.871	0.495	0.076	3.801	0.014	0.703

## Data Availability

Not applicable.

## Supplementary Materials

The following supporting information can be downloaded at: https://www.mdpi.com/article/10.3390/ijms241914933/s1.

## Abbreviations

**Notation**	
$C_{B,exp}$	Experimental purity of *L*-menthol in the final product, dimensionless
$C_{B,the}$	Calculated purity of *L*-menthol in the final product, dimensionless
$L_{0\;}$	Mass of the initial liquid feed, g
$L_{n\;}$	Mass of the liquid out of stage n, g
${P_{n}}_{}$	Pressure in stage n, Pa
$P_{j}^{sat}$	Saturated vapor pressure of component-j, Pa
$P_{tri,j}$	Triple-point pressure of component-j, K
R	Ideal gas constant, 8.314 J/mol−K
$S_{n}$	Mass of the solid solution enriched with *L*-menthol formed in stage n, g
$S_{tot,n}$	Total amount of the solid solution enriched with *L*-menthol formed from stage 1 to stage n, g
$T_{n}$	Temperature in stage n, K
$T_{b,j}$	Boiling temperature of component-j, K
$T_{m,j}$	Melting temperature of component-j, K
t	Time, s
$V_{n}$	Mass of the vapor formed in stage n, g
$V_{tot,n}$	Total amount of vapor formed and removed from stage 1 to stage n, g
$W_{f,exp}$	Measured weight of the final product at the end of TPC, g
$W_{f,the}$	Calculated weight of the final product at the end of TPC, g
${\left(X_{j}\right)}_{0}$	Initial mole fraction of component-j in the liquid feed, dimensionless
${\left(X_{j}\right)}_{n}$	Mole fraction of component-j in the remaining liquid in stage n, dimensionless
${\left(Y_{j}\right)}_{n}$	Mole fraction of component-j in the vapor phase formed in stage n, dimensionless
${\left(Z_{j}\right)}_{n}$	Mole fraction of component-j in the solid solution formed in stage n, dimensionless
${\bar{Z}}_{B,the}$	Average mole fraction of component-j in the solid solution at the end of T, dimensionless
${\bar{Z}}_{B,exp}$	Experimental purity of *L*-menthol for the final product at the end of the sweating experiment, dimensionless
$∆H_{m,j\;}$	Heat of melting for component-j (>0), J/mol
${∆H}_{V,j\;}$	Heat of vaporization for component-j (>0), J/mol
${∆L}_{exp}$	Amount of liquid removed at the end of the sweating experiment, g
**Greek Letters**	
$\gamma_{j}$	Activity coefficient of component-j in liquid, dimensionless
**Subscript**	
0	In the initial feed
f	At the end of TPC
n	In stage n
N	In the last stage
