# Supplementary material for: The Influence of the Solid Solution Formation on Purification of L-Menthol from the Enantiomer Mixture by Three-Phase Crystallization
Source: Int J Mol Sci. 2023 Oct 5;24(19):14933. doi: 10.3390/ijms241914933 (PMC10573351; doi:10.3390/ijms241914933)
Supplement: Supplementary file 1 [file ijms-24-14933-s001.zip › ijms-2623739-supplementary.pdf]

Supplementary Materials

Table S1. The calculated results of TPC for $L_0 = 5$ g feed with $(X_B)_0 = 0.93$.

**n**	$\boldsymbol{T}$(°C)	$\boldsymbol{P}$**(Pa)**	$(\boldsymbol{X_B})_{\boldsymbol{n}}$	$(\boldsymbol{Z_B})_{\boldsymbol{n}}$	$\boldsymbol{L_n}$**(g)**	$\boldsymbol{S_n}$**(g)**	$\boldsymbol{S_{tot,n}}$**(g)**	$\boldsymbol{V_n}$**(g)**	$\boldsymbol{V_{tot,n}}$**(g)**
0	39.7	24.5	0.930	0.981	5	0	0	0	0
1	38.7	22.1	0.908	0.971	2.913	1.761	1.761	0.326	0.326
2	37.7	19.9	0.886	0.960	1.885	0.867	2.628	0.160	0.486
3	36.7	18.0	0.864	0.949	1.309	0.486	3.114	0.090	0.576
4	35.7	16.2	0.841	0.939	0.956	0.298	3.412	0.055	0.631
5	34.7	14.6	0.819	0.928	0.726	0.195	3.607	0.036	0.667
6	33.7	13.1	0.797	0.917	0.568	0.134	3.740	0.025	0.692
7	32.7	11.8	0.775	0.907	0.455	0.095	3.836	0.018	0.710
8	31.7	10.6	0.753	0.896	0.371	0.070	3.906	0.013	0.723
9	30.7	9.5	0.731	0.886	0.308	0.053	3.959	0.010	0.733
10	29.7	8.5	0.709	0.875	0.260	0.041	4.000	0.008	0.741

Table S2. The calculated results of TPC for $L_0 = 5$ g feed with $(X_B)_0 = 0.95$.

n	$\boldsymbol{T}$(℃)	$\boldsymbol{P}$(Pa)	$(\boldsymbol{X_B})_{\boldsymbol{n}}$	$(\boldsymbol{Z_B})_{\boldsymbol{n}}$	$\boldsymbol{L_n}$(g)	$\boldsymbol{S_n}$(g)	$\boldsymbol{S_{tot,n}}$(g)	$\boldsymbol{V_n}$(g)	$\boldsymbol{V_{tot,n}}$(g)
0	40.6	26.9	0.950	0.991	5	0	0	0	0
1	39.6	24.3	0.928	0.98	2.502	2.108	2.108	0.390	0.390
2	38.6	21.9	0.906	0.97	1.477	0.865	2.973	0.160	0.550
3	37.6	19.7	0.884	0.959	0.964	0.433	3.406	0.080	0.630
4	36.6	17.8	0.861	0.948	0.673	0.245	3.651	0.045	0.675
5	35.6	16.0	0.839	0.938	0.494	0.151	3.803	0.028	0.703
6	34.6	14.4	0.817	0.927	0.376	0.099	3.902	0.018	0.721
7	33.6	13.0	0.795	0.916	0.295	0.069	3.971	0.013	0.734
8	32.6	11.7	0.773	0.906	0.237	0.049	4.020	0.009	0.743
9	31.6	10.5	0.751	0.895	0.194	0.036	4.056	0.007	0.750
10	30.6	9.4	0.729	0.884	0.161	0.027	4.083	0.005	0.755
11	29.6	8.4	0.706	0.874	0.136	0.021	4.105	0.004	0.759

Table S3. The calculated results of TPC for $L_0 = 5$ g feed with $(X_B)_0 = 0.97$.

n	T(℃)	P(Pa)	$(X_B)_n$	$(Z_B)_n$	L_n(g)	S_n(g)	$S_{tot,n}$(g)	V_n(g)	$V_{tot,n}$(g)
0	41.6	29.7	0.970	1.000	5	0	0	0	0
1	40.6	26.9	0.948	0.990	1.881	2.632	2.632	0.487	0.487
2	39.6	24.3	0.926	0.979	0.960	0.778	3.409	0.144	0.631
3	38.6	21.9	0.904	0.969	0.573	0.327	3.736	0.060	0.691
4	37.6	19.7	0.882	0.958	0.376	0.166	3.902	0.031	0.722
5	36.6	17.8	0.859	0.947	0.264	0.095	3.996	0.018	0.740
6	35.6	16.0	0.837	0.937	0.195	0.059	4.055	0.011	0.751
7	34.6	14.4	0.815	0.926	0.149	0.039	4.094	0.007	0.758
8	33.6	13.0	0.793	0.915	0.117	0.027	4.121	0.005	0.763
9	32.6	11.7	0.771	0.905	0.094	0.019	4.140	0.004	0.767
10	31.6	10.5	0.749	0.894	0.077	0.014	4.154	0.003	0.770
11	30.6	9.4	0.727	0.884	0.064	0.011	4.165	0.002	0.772
12	29.6	8.4	0.704	0.873	0.054	0.008	4.174	0.002	0.774
